# Synthesis and Performances of Phase Change Microcapsules with a Polymer/Diatomite Hybrid Shell for Thermal Energy Storage

**DOI:** 10.3390/polym10060601

**Published:** 2018-05-30

**Authors:** Yanli Sun, Rui Wang, Xing Liu, Erqing Dai, Bo Li, Shu Fang, Danyang Li

**Affiliations:** 1 School of Textiles, Tianjin Polytechnic University, No. 399 Bin Shui Xi Road, Xi Qing District, Tianjin 300387, China; yls198959@163.com (Y.S.); liuxing@tjpu.edu.cn (X.L.); 1988li01bo22@163.com (B.L.); fairyfangshu@gmail.com (S.F.); strawberry4173@163.com (D.L.); 2 Key Laboratory of Advanced Textile Composites (Tianjin Polytechnic University), Ministry of Education, Tianjin 300387, China; 3 Affiliated Hospital of Logistics University of People’s Armed Police Force, Tianjin 300162, China; 13502136445@163.com

**Keywords:** phase-change microcapsules, mechanical behavior, Atomic Force Microscopy, Young’s modulus, diatomite

## Abstract

The mechanical behavior of phase-change microcapsules (microPCMs) is of vital significance for practical applications in thermal energy storage. Hence, a new type of microPCMs based on an n-octadecane (C18) core and a melamine-urea-formaldehyde (MUF)/diatomite hybrid shell was developed through in situ polymerization. Based on SEM micrographs, most microPCMs exhibited a nearly spherical and smooth microstructure, with broadened particle size distributions. It was confirmed by Fourier transform infrared (FTIR) that successful polymerization of diatomite into the microPCMs occurred, and that additional diatomite had no effect on the core coated by the shell. In addition, the results of the differential scanning calorimeter (DSC) and Atomic Force Microscopy (AFM) demonstrated that the mechanical properties of the microPCMs were remarkably improved by the addition of a moderate amount of diatomite, but that the heat enthalpy and encapsulated efficiency (η) decreased slightly. The incorporation of 2 wt % diatomite resulted in the average Young’s modulus of microPCMs, which was 1.64 times greater than those of microPCMs without diatomite. Furthermore, the melting and crystallization enthalpies and the encapsulated efficiency of the microPCMs were as high as 237.6 J/g, 234.4 J/g and 77.90%, respectively. The microPCMs with a polymer/diatomite hybrid shell may become the potential materials in the application of thermal energy storage.

## 1. Introduction

In recent decades, with the rapid development of the economy, energy consumption and demand has increased quickly. However, conventional energy resources are limited, and their application has already led to climate changes, such as air pollution and the greenhouse effect. Therefore, the efficient use of clean and renewable energy resources is a popular research topic. Phase-change materials (PCMs) have been known to be excellent candidates for thermal energy storage because PCMs can absorb and release latent heat within a narrow temperature range during the phase-transition process, and, especially, as they can be reused to reduce energy waste [1,2]. Paraffin waxes, as solid-liquid PCMs, are widely applied because of their merits [3], such as their small amount of supercooling, nontoxicity, non-corrosiveness, chemical stability, and high latent heat energy. However, Paraffin waxes show some inherent drawbacks, such as low thermal conductivity and leakage during the melting process, which affects their practical application. To prevent the leakage, controlling the volume change of PCMs during the phase change and increasing the heat-transfer ability can be used as methods to avoid the drawbacks of PCMs. The methods include impregnating PCMs into a polymer matrix [4,5,6], use of a porous or layered material [7,8,9], and microencapsulation PCMs. Phase-change microcapsules (microPCMs) have been developed and used. MicroPCMs, with typical core-shell structure, have PCMs as the core surrounded by organic or inorganic material as the shell. MicroPCMs have been used in solar energy storage [10,11], air conditioning [12], building energy conservation [13], and in thermal-regulating fibers and textiles [14].

MicroPCMs with organic shells, such as urea-formaldehyde resin [15], melamine-formaldehyde resin [16], poly (methyl methacrylate) resin [17], and polyuria [18], have been widely studied and applied due to the good structural flexibility and sealing tightness of organic shells. However, there are still some drawbacks of microPCMs with organic shells, such as flammability, poor thermal stabilities, low mechanical strength, and thermal conductivity. Specifically, the mechanical behavior of microPCMs is a key factor in their practical application. Excellent mechanical behavior is not easily satisfied using single organic shells. Therefore, some studies chose to use inorganic shells instead of organic shells. Wang et al. [19] compounded microPCMs, based on paraffin-based binary cores and calcium carbonate shell, via the self-assembly method. Jiang et al. [20] successfully prepared microencapsulated PCMs with TiO_2_/Fe_3_O_4_ hybrid shells as thermoregulatory enzyme carriers. Zhang et al. [21] fabricated microencapsulated PCMs based on an n-octadecane (C18) core and silica shell through interfacial polycondensation. As a result, the microPCMs with inorganic shells present excellent mechanical strength. Nevertheless, the low flexibility leads to poor endurance during practical applications. To overcome the identified shortcomings, several researchers have focused on using an organic-inorganic hybrid shell as it can obtain a synergetic combination of unique properties that exploit the mechanical properties of inorganic shells and the flexibility of organic shells. Inorganic nanoparticles are usually added to microPCMs, such as nanosilver [22], silicon dioxide (SiO_2_) [23], aluminum oxide (Al_2_O_3_) [24], graphene [25], and carbon nanotubes (CNTs) [26].

In our previous work [27], we used O_2_-plasma-modified multiwalled carbon nanotubes (CNTs) to modify the C18-based microPCMs through in situ polymerization. Meanwhile, two different addition methods, the addition of modified CNTs into the emulsion system or into the polymer system, were compared and examined. The results indicated that the mechanical properties of the microPCMs were remarkably improved by the addition of a moderate amount of modified CNTs into the polymer system. The excellent properties of CNTs are beneficial for enhancing the mechanical performance of microPCMs, but the cost of CNTs is slightly higher and pretreatment is required to increase its dispensability in the prepolymer.

Diatomite consists of fossilized remains of diatoms, which is a type of hard-shelled protest [28]. Diatomite possesses high porosity, certain rigidity, and inertness [29]. The main chemical composition of diatomite is silica and diatomite is abundantly available in China, as well as other countries. The price of diatomite is cheap and its source is rich and, thus, diatomite can be used as inorganic nanoparticles and added to microPCMs. However, little work has chosen diatomite to prepare microPCMs with organic-inorganic hybrid shells. In this study, we successfully synthesized microPCMs based on a C18 core and a melamine-urea-formaldehyde (MUF)/diatomite hybrid shell through in situ polymerization, and aimed to achieve improvements in the mechanical properties of microPCMs. The effects of the addition of diatomite on the microstructure, particle size distribution, thermal properties, and mechanical properties of microPCMs have been discussed.

## 2. Experimental

### 2.1. Materials

C18 was used as a core material and was purchased from Alfa Aesar(China) Chemicals, Co., Ltd. (Shanghai, China)-. Melamine (M), urea (U), and a 37% formaldehyde solution (F) were used as shell materials. M and U were supplied by Tianjin Guangfu Fine Chemical Institution (Tianjin, China), F was supplied by Sigma Aldrich, St. Louis, MO, USA. Sodium dodecyl sulfate (SDS) was used as an emulsifying agent and was obtained from Tianjin Kemiou Chemical Reagent Co., Ltd. (Tianjin, China). Citric acid and triethanolamine were used to control the pH and were acquired from Tianjin Guangfu Fine Chemical Institution (China). Diatomite (average particle size 1–3 μm) was provided by Changbai Mountain Korean Autonomous County Xinxin Diatomite Co., Ltd. (Baishan, China).

### 2.2. Synthesis of microPCMs with MUF/Diatomite Hybrid Shell

**Figure 1:**
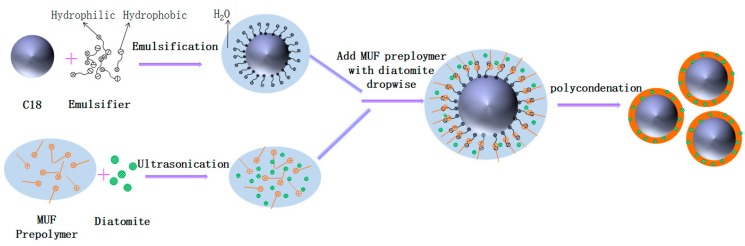
Synthetic scheme for phase-change microcapsules (microPCMs) with melamine-urea-formaldehyde (MUF)/diatomite hybrid shells via in situ polymerization.

Microencapsulated C18, with a MUF/diatomite hybrid shell, were synthesized by in situ polymerization. As shown in Figure 1, the microencapsulation procedure included three key stages: (1) Preparation of the MUF prepolymer and addition of diatomite, (2) C18 emulsification, and (3) polymerization of the shell materials.

Firstly, 3.81 g of melamine, 6.89 g of aqueous formaldehyde, and 70 mL of distilled water were mixed in a beaker. The pH value of the mixture was adjusted to 8.5 with triethanolamine. The reaction was continued at 70 °C for 20 min under mechanical stirring. Then, the system was cooled to room temperature and 0.92 g of urea was added to the MUF prepolymer. A certain amount of diatomite were added to the MUF prepolymer solution and ultrasonically dispersed for 10 min. Secondly, 15 g of C18 was emulsified at 1000 rpm in a 10 wt % SDS aqueous solution at 40 °C for 30 min to a generate a stable oil-in-water (O-W) emulsion. Thirdly, the pH value of the emulsion was controlled at 5 with 13 wt % citri acid solution, the emulsion was continuously stirred at 300 rpm, and the temperature was adjusted to 75 °C. The MUF preploymer dispersing diatomite was, subsequently, added dropwise to the emulsion and the system continued to react for 3.5 h. Finally, microPCMs, with MUF/diatomite hybrid shells, were obtained by filtration and dried at room temperature. Table 1 shows the specific process parameters.

### 2.3. Characterization

#### 2.3.1. Fourier Transform Infrared

Fourier transform infrared (FTIR) spectra characterized the chemical structure of the C18, MUF shell. MicroPCMs were obtained using a FTIR spectroscopy (TENSOR37, Bruker, Saarbrücken, Germany) in the range of 4000 to 500 cm^−1^ at a resolution of 4 cm^−1^ using KBr pellets at room temperature.

#### 2.3.2. Scanning Electron Microscopy Observation and Particle Size Analysis

The surface morphologies and the wall thickness of the microPCMs were observed using a field emission scanning electron microscope (FESEM, S-4800, Hitachi, Tokyo, Japan) at an accelerating voltage of 10.0 kV. The specimens were sprayed with gold prior to observation. Evaluation method of wall thickness is as follows: Firstly, the microPCMs were adhered to a sample platform with a conductive adhesive and, then, the microPCMs on the sample platform were cut by a sharp blade in a liquid nitrogen environment. Finally, the cross-section of the microPCMs was observed using FESEM and measured by Image-Pro Plus analysis software (6.0). The particle size distribution was examined with Image-Pro Plus analysis software and over 200 microPCMs were counted.

#### 2.3.3. Differential Scanning Calorimeter

The phase-change properties of the C18 and the microPCMs were analyzed using a differential scanning calorimeter (DSC) (Netzsch 204F1, Netzsch Group, Bavaria, Germany) in the temperature range of −20 to 80 °C at a heating/cooling rate of 5 °C/min under a nitrogen atmosphere.

#### 2.3.4. Thermalgravimetric Analysis

The thermal stability of the C18 and the microPCMs was performed using a thermalgravimetric analysis (TG) (Netzsch STA 409 PC/PG TG–DTA, Netzsch Group, Bavaria, Germany) in the temperature range of 25 to 600 °C at a heating/cooling rate of 10 °C/min under a nitrogen atmosphere.

#### 2.3.5. Mechanical Properties

The mechanical properties of the microPCMs were examined using Atomic Force Microscopy (AFM) (CSPM5500, Benyuan, Beijing, China) [15,30]. The Young’s modulus (E) of the samples was calculated from the force curve measured by AFM. The sample was glued on a disk with epoxy, and then spherical and clean particles were chosen to be tested using the optical system. AFM topographic images were acquired using the non-contact mode, and the force curve was obtained using the contact mode. The ranges of the scan areas was 2 μm × 2 μm and scan frequency was 1 Hz. The probe (AC240TS, Olympus, Tokyo, Japan) material was Si, with an elastic modulus of 150 GPa, a Poisson’s ratio of 0.17, and an elastic coefficient of the probe of 1.73 N/m. The shape of the cantilever was rectangle and the tip had a 3-sided shape.

**Figure 2:**
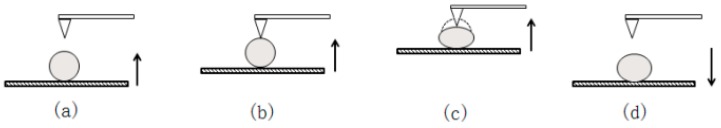
The test process of phase-change microcapsules’ (microPCMs) force curve: (**a**) the sample starts to rise, (**b**) the sample begins to contact with the probe, (**c**) maximum deformation of cantilever, and (**d**) the sample drop.

The test process of the sample force curve is depicted in Figure 2a–d. Figure 2a shows as the piezoelectric ceramic voltage increases, the sample starts to rise, with no contact between the probe and the sample. The sample begins to contact with the probe and continues to rise (Figure 2b). Then, as can be seen in Figure 2c, the cantilever deforms as the sample continuously rises. According to the deformation of the cantilever, the load, *f*, can be calculated. The sample has a certain deformation under the action of *f*. After the deformation of the cantilever reaches a certain point, that is *f* reaches a certain value, the sample starts to be withdrawn and unloaded continuously (Figure 2d).

## 3. Results and Discussion

### 3.1. FTIR Spectra

**Figure 3:**
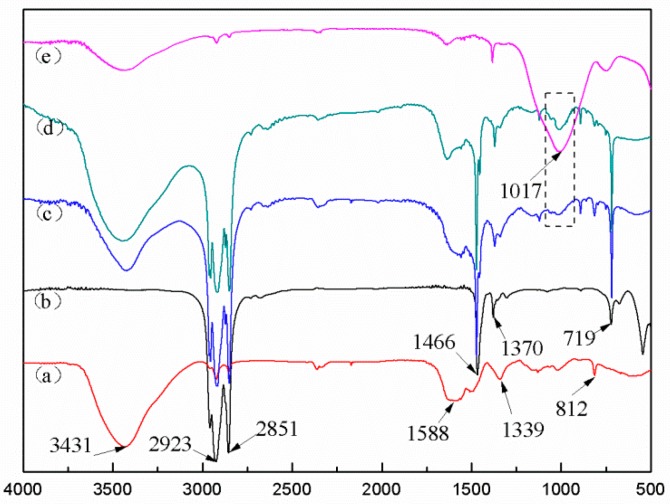
Fourier transform infrared (FTIR) spectra of C18 and phase-change microcapsules (microPCMs): (a) Melamine-urea-formaldehyde (MUF) shell, (b) C18, (c) S0, (d) S4, and (e) diatomite.

Figure 3 presents the FTIR spectra of the MUF shell, C18, microPCMs (S0 and S4), and diatomite. In the spectrum of the MUF shell (Figure 3a), an absorption peak at 3431 cm^−1^ was identified as the stretching vibrations of O–H and N–H, the peaks at 1588 and 1339 cm^−1^ corresponded to the stretching vibrations of C–N and C=N, and the peak at 812 cm^−1^ ascribed to the bending vibration of the triazine ring. It can be seen in Figure 3b that the two strong absorption peaks at 2923 and 2851 cm^−1^ were caused by the C–H stretching vibrations, the absorbance at 1466 cm^−1^ was attributed to the –CH_2_ and –CH_3_ deformation vibrations, and that the –CH_2_ in-plane rocking vibrations were found at approximately 719 cm^−1^. After microencapsulation, the characteristic peaks of the MUF shell and C18 appeared in the spectrum of the microPCMs (S0 and S4) without new peaks, and the location of the peaks maintained the same level. There were just physical combinations existing between the core and the shell. A characteristic peak at 1017 cm^−1^ in the diatomite spectrum (Figure 3e) was ascribed to the stretching vibration peak of Si–O–Si; this peak also appeared in the spectrum of the S4. It can be inferred that successful polymerization of diatomite into the microPCMs and addition of diatomite had no effect on the core coated by the shell.

### 3.2. Morphology, Wall Thicknesses, and Particle Size Distribution

**Figure 4:**
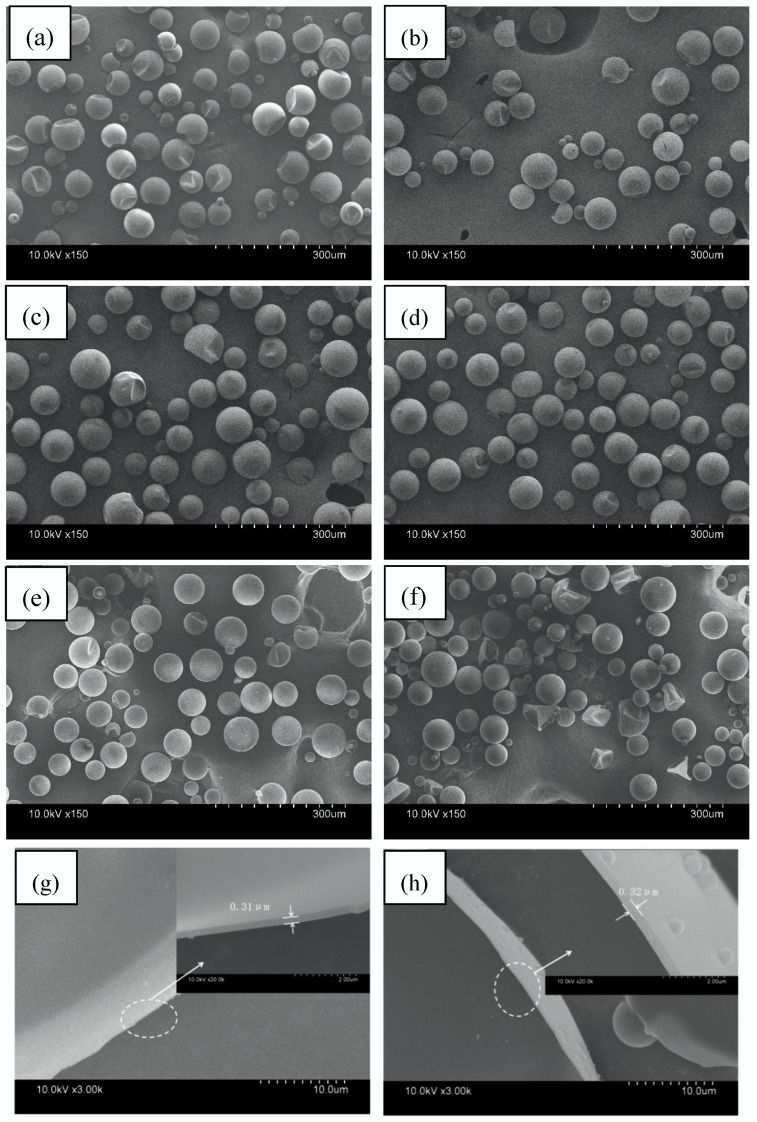
Scanning electron microscopy images of phase-change microcapsules (microPCMs) and damaged microPCMs with different amounts of diatomite added: (**a**–**g**) S0, (**b**) S1, (**c**) S2, (**d**) S3, (**e**–**h**) S4, and (**f**) S5.

Figure 4 shows the microstructures and surface morphology of the microPCMs (S0, S1, S2, S3, S4 and S5), and the wall thicknesses of the S0 and S4. It can be observed that most the microPCMs were close to spherical, which had smooth surfaces and dispersed without any agglomeration. Meanwhile, the SEM micrographs show that the surfaces of the microPCMs had some dimples. These dimples were ascribed to the space shrinkage of the C18 phase, changing from the melting state to the crystal state. Furthermore, it is notable that the small dimples became fewer and more inconspicuous as the amount of diatomite increased (Figure 4a–e). However, when diatomite continued to increase, ruptured microPCMs appeared (Figure 4f). This may suggest that the addition of a moderate amount of diatomite could enhance the mechanical strength of the shell and will hardly deform [31]. Excessive amounts of diatomite led to an agglomeration of particles and had a negative influence on the adsorption of the shell on the surface of the core [32], which was not beneficial to the core coated by the shell.

Figure 4g–h shows the wall thickness of the microPCMs (S0 and S4), which is approximately 0.31 and 0.32 μm, respectively. This indicates that the addition of diatomite had less of an effect on the wall thickness.

**Figure 5:**
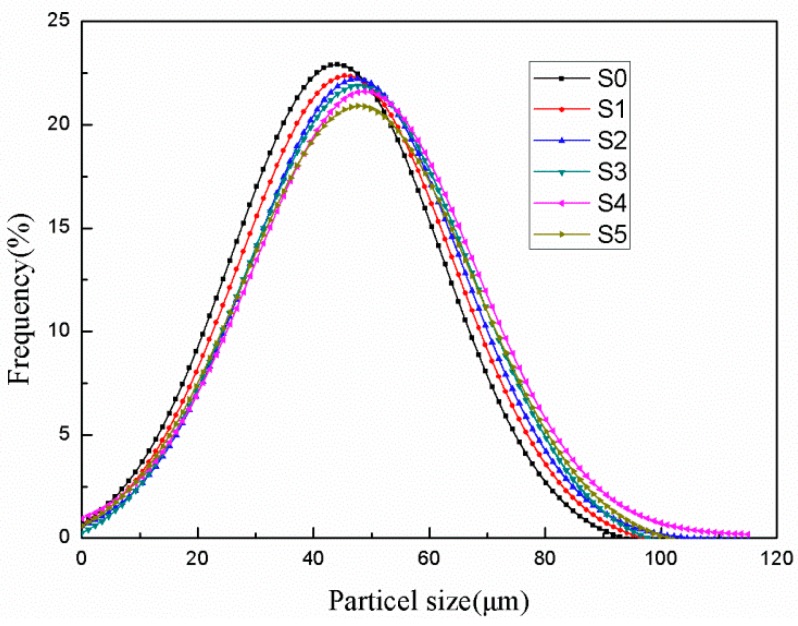
Particle size distributions of phase-change microcapsules (microPCMs) with different amounts of diatomite.

Figure 5 presents the particle size distribution of the microPCMs and Table 2 lists the average particle size. It can be found that the size of the samples was mainly distributed in the range of 10~100 μm, and had broadened particle size distributions. The average particle size of S0 was approximately 40.26 μm, whereas the average particle sizes of S1, S2, S3, S4, and S5 were measured to be 41.31, 42.34, 41.96, 42.81, and 42.56 μm (±2 μm, the measuring error), respectively. There was almost no difference between the average particle sizes of the microPCMs with diatomite and the microPCMs without diatomite. This showed that the adding of diatomite had little impact on the particle size of the microPCMs. The results agree with our previous work [27].

### 3.3. Phase-Change Properties and Thermal Storage Capability

**Figure 6:**
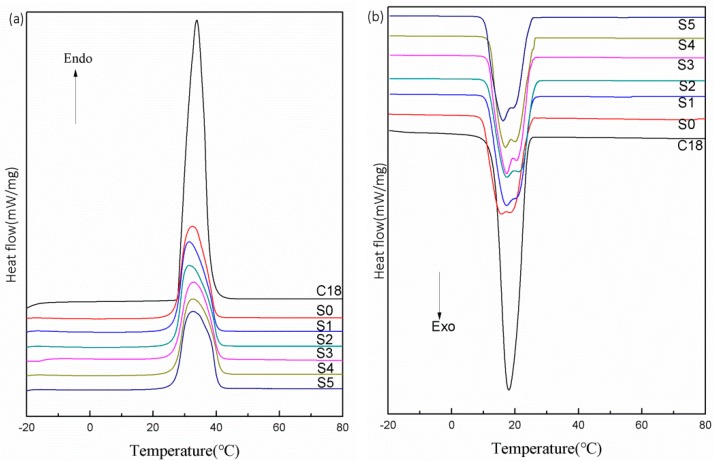
Differential scanning calorimeter (DSC) thermograms of C18, S0, S1, S2, S3, S4, and S5: (**a**) Heating thermograms and (**b**) cooling thermograms.

The phase-change behaviors of the C18 and microPCMs (S0, S1, S2, S3, S4 and S5) were evaluated by DSC and the resulting thermograms are presented in Figure 6. The melting and crystallization parameters obtained from DSC are listed in Table 2. Compared with the C18, the melting temperature (T_m_) of the microPCMs moved towards lower temperatures and the crystallization temperature (T_c_) of the microPCMs shifted to slightly higher temperatures, as shown in Figure 6 and Table 2. Similar phenomena has been reported elsewhere [33]. This result was ascribed to two reasons; one was that the C18 was microencapsulated to increase the surface areas and the other was that the motion was confined inside a narrow space. Hence, the heat/cool transportation became faster from the outside to the core.

It is observed, in Figure 6, that C18 presents an endothermic peak in the melting process and an exothermic peak in the crystallization process, while an endothermic peak and multiple exothermic peaks are presented in the phase-change process of the microPCMs. Moreover, the multiple exothermic peaks become more obvious at increasing amounts of diatomite. There are two PCMs crystallizations in the cooling process, namely, homogeneous and heterogeneous crystallization [34,35]. When microencapsulated, C18 was coated by the MUF shell and the inner shell can act as a nucleating agent, which generates heterogeneous nucleation crystallization and multiple peaks appear in the solidifying curves. Diatomite added to the shell can also play a role as nuclei and the heterogeneous crystallization peak increased and, thus, the above results appeared [36]. These facts also revealed that the addition of diatomite improved the heterogeneous nucleation crystallization of C18.

In addition, the heat enthalpy and encapsulated efficiency (η) are also crucial parameters to evaluate the microPCMs. The specific values are provided in Table 2. The encapsulated efficiency, η, was evaluated using the following formula [37]:

(1) $$\eta =\frac{\Delta H_{m,microPCMs}+\Delta H_{c,microPCMs}}{\Delta H_{m,PCMs}+\Delta H_{c,PCMs}}\times 100\%$$

where Δ*H_m,microPCMs_* and Δ*H_c,microPCMs_* are the melting enthalpy and crystallization enthalpy of the microPCMs; and Δ*H_m,PCMs_* and Δ*H_c,PCMs_* are the melting enthalpy and crystallization enthalpy of C18, respectively.

Obviously, the melting enthalpy (*ΔH_m_*) and the crystallization enthalpy (*ΔH_c_*) of the microPCMs were dramatically lower in comparison to the C18, and the *ΔH_m_*, *ΔH_c_*, and η were slightly reduced with an increase in diatomite contents, as seen in Table 2. In the DSC scanning temperature range, the MUF shell and diatomite did not undergo any phase-change, with only the C18 being able to store and release the latent heat. It is notable that the core-shell weight ratio was a key factor influencing heat enthalpy. When 3 wt % of diatomite was added, the heat enthalpy and η still reached more than 225 J/g and 75%, respectively, which satisfied the applied requirements for heat storage and exchange.

### 3.4. Thermal Stability

**Figure 7:**
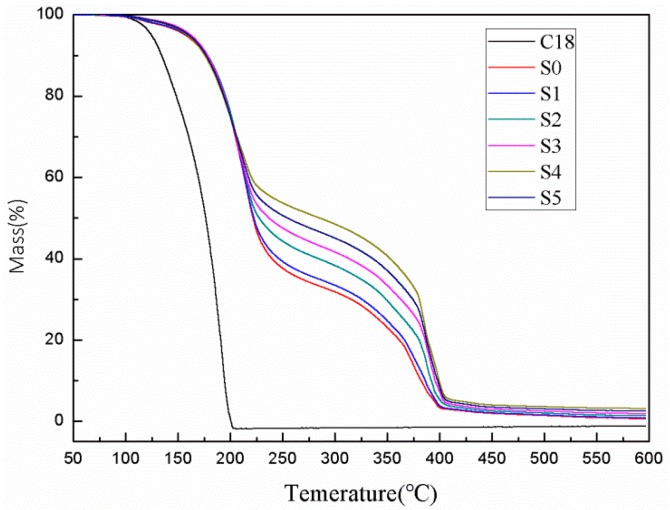
Thermogravimetry spectra of C18, S0, S1, S2, S3, S4, and S5.

The thermal decomposition properties of the C18 and microPCMs (S0, S1, S2, S3, S4, and S5) were studied by TG and the resulting thermograms are presented in Figure 7. From Figure 7, it is obvious that the C18 exhibits a typical one-step thermal decomposition process. The C18 started to lose weight at 130 °C and the degradation process ended up at about 200 °C. By contrast, all the microPCMs samples show a two-stage thermal decomposition process. The first step of weight loss, in the range of 180–350 °C, was caused by vaporization of the C18 from the microPCMs and its further degradation. The second step of weight loss, at about 350–420 °C, was attributed to the MUF shell pyrolysis. We can easily find that the onset degradation temperature of the microPCMs was almost 50 °C higher than that of the C18. This means that the shell provided an effective barrier and could delay the rapid C18 decomposition. Furthermore, the starting thermal degradation temperature was delayed to 181.2 °C for S0 and to 182.3, 183.5, 184.3, 184.7, and 183.6 °C for S1, S2, S3, S4, and S5, respectively. Thus, the above results indicate that the starting thermal degradation temperature had no obvious change as the addition of diatomite and the thermal stability was almost unaffected by the hybrid shell.

### 3.5. Mechanical Properties

**Figure 8:**
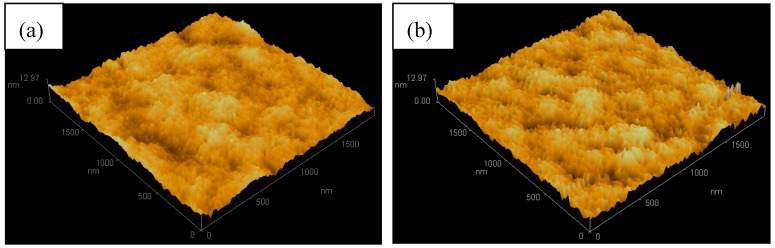
Atomic Force Microscopy (AFM) images of the surfaces of the phase-change microcapsules (microPCMs): (**a**) S0 and (**b**) S4.

The surface profile of the S0 and S4 were further elucidated by AFM, as shown in Figure 8. The S0 exhibits rather smooth outlines. However, the surface of the S4 presents some small protuberances due to the embedded diatomite.

In order to further confirm that adding diatomite could improve the mechanical properties of microPCMs, the mechanical properties of the microPCMs were tested by AFM. The effect of the addition of diatomite on the mechanical properties of the microPCMs was investigated by comparison with *E*. *E* was obtained by analyzing the force curve and calculated by using the Hertz model [30]. The specific calculation formula is as follows:

(2) $$f=\frac{\pi }{2}\frac{E}{1-v^{2}}\tan\alpha \;\delta^{2}$$

where *f* is the force exerted by the probe on the microcapsules, nN; *v* is the Poisson ratio with a value of 0.33; α is the probe half angle with a value of 36°; and δ is the sample penetration.

**Figure 9:**
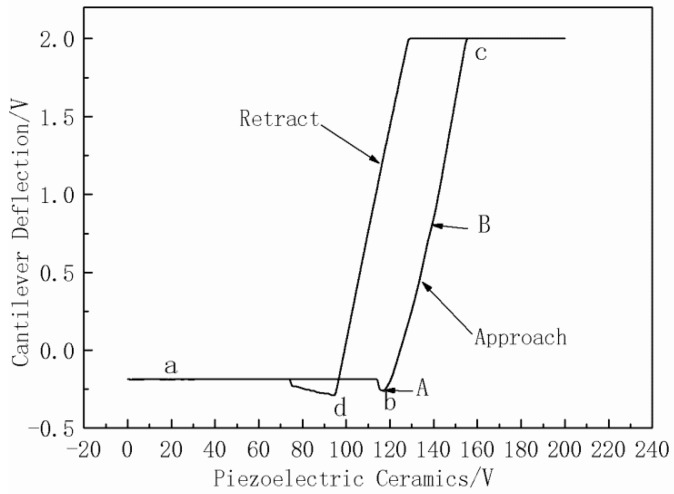
The force curve of prototype.

A hard, smooth, and non-deformable material was selected as a standard sample for testing, and the *f* and δ could be calculated by analyzing the standard sample. We choose the mica film as the standard sample, and the force curve of the mica film is shown in Figure 9. The *x*-axis is the displacement of the piezoelectric ceramics in the Z direction, which presents the rising height of the sample; the *y*-axis presents the cantilever deflection; and a, b, c, and d represents the four processes of the force curve test. The sample begins to contact with the probe at point b and the cantilever deforms and loads the sample.

When the probe starts to touch the standard sample (A) and until the cantilever deflection is 1 V (B), the rising height of the standard sample is *H*. *H* is expressed in Equation (3), where *Z* is the scaling coefficient of the scanner, with a value of 10.811 V/nm; *X_A_* and *X_B_* is the coordinate point corresponding to the *X* axis, respectively.

(3) H=(XA−XB)×Z

*f* is calculated as in Equation (4):

(4) f=k·H

Similar force curves could be obtained by testing microPCMs samples. When the probe starts to touch the microPCMs sample (A’) and until the cantilever deflection is 1 V (B’), the rising height of the microPCMs sample is *H*’ and the force acting on the microPCMs samples is *f*. *H*’ is acquired by Equation (3) and the δ due to the exerted *f* is evaluated as Equation (5). Then, E could be obtained using the Hertz model. 

(5) $$\delta =H-H^{\prime }$$

Table 3 gives the maximum, minimum, and average values of the effective test results of the microPCMs E. As seen in Table 3, the dates measured by this method show large discreteness. Similar results has been obtained in reference [15,30]. The wide dispersion of the test results were mainly related to the test method. The test of the AFM force curve is affected by various factors, such as the surface morphologies and defects of the microPCMs, probe stiffness, loading rate, and indentation depth, which can result in a significant dispersion of the test results. However, by comparing the average value of *E*, we found that the Young’s modulus of the microPCMs with diatomite increased significantly. The average Young’s modulus of the microPCMs with 2 wt % diatomite (S4) was 942.85 MPa, which was 1.64 times greater than those of the microPCMs without diatomite (S0). This value indicates that the assembled diatomite could change the Young’s modulus of the MUF shell and significantly strengthen the microPCMs, even in relatively low mass percentages. Diatomite was evenly dispersed into the shell of the microPCMs. When the microPCMs was subjected to external force, the diatomite could produce a stress concentration effect, which absorbed energy and prevented further deformation and the rupture of the microPCMs. A similar phenomenon has been observed in reference [27,38].

Additionally, we discovered that the Young’s modulus increased initially and then decreased slightly with increasing diatomite content. It was very difficult to have microPCMs with excellent mechanical properties when the diatomite content became higher. Extensive agglomeration of diatomite particles occurred in the MUF shell when the amount of diatomite reached a value, leading to the uneven distribution of diatomite in the shell and poor interfacial adhesion with the shell. As a result, some formed shell might have loose structures and rupture easily. These results suggest that microPCMs incorporated with a moderate amount of diatomite could improve the mechanical properties and exhibits a good potential application for energy storage.

## 4. Conclusions

A novel kind of microencapsulated PCMs with MUF/diatomite hybrid shells were successfully synthesized through in situ polymerization. The effects of diatomite particles and contents on the FTIR, microstructure, particle size distribution, thermal properties, and mechanical properties of microPCMs were investigated. Results showed that most of the microPCMs had a spherical and smooth microstructure, were dispersed without any agglomeration, and had broadened particle size distributions. Moreover, the addition of diatomite had no effect on the core coated by the shell. The investigation of the heat storage performance of the microPCMs indicated that an increase in diatomite content could slightly reduce the heat enthalpy and η. According to the result of the mechanical properties, the incorporation of diatomite greatly enhanced the Young’s modulus of the MUF shell and significantly strengthened the microPCMs. When 2 wt % diatomite was incorporated, the melting and crystallization enthalpies and the encapsulated efficiency of the microPCMs were as high as 237.6 J/g, 234.4 J/g and 77.90%, respectively. Additionally, the average Young’s modulus of S4 was 942.85 MPa, which was 1.64 times greater than those of the microPCMs without diatomite. The microPCMs with a polymer/diatomite hybrid shell exhibited a high heat enthalpy and good mechanical properties, which is a promising prospect in the fields of thermal energy storage, temperature-regulating textiles, and building energy conservation.

## Figures and Tables

**Table 1:** Preparation of phase-change microcapsules (microPCMs) with melamine-urea-formaldehyde (MUF)/diatomite hybrid shells.

	microPCMs	S0	S1	S2	S3	S4	S5	
emulsion system	N-octadecane (g)	15	15	15	15	15	15	
	SDS (wt %)	10	10	10	10	10	10	
	Distilled water (mL)	90	90	90	90	90	90	
Pre-polymer system	Melamine (g)	3.81	3.81	3.81	3.81	3.81	3.81	
	Formaldehyde (g)	6.89	6.89	6.89	6.89	6.89	6.89	
	Urea (g)	0.92	0.92	0.92	0.92	0.92	0.92	
	Distilled water(mL)	70	70	70	70	70	70	
							
	Diatomite (%)	0	0.5	1	1.5	2		3

**Table 2:** The average particle size and phase change properties of the C18 and phase-change microcapsules (microPCMs), incorporated with different amounts of diatomite.

Samples	Average Particle Size (μm)	*T_m_* (°C)	*ΔH_m_* (J/g)	*T_c_* (°C)	*ΔH_c_* (J/g)	η (%)
C18	0	27.5	307.1	25.5	298.8	0
S0	40.26	26.9	250.1	25.8	255.6	83.46
S1	41.31	26.8	245.8	25.9	248.1	81.52
S2	42.34	26.9	240.2	25.8	241.4	79.49
S3	41.96	26.7	243.9	25.9	241.1	80.05
S4	42.81	26.8	237.6	25.8	234.4	77.90
S5	43.56	26.8	227.3	25.9	228.2	75.18

**Table 3:** The Young’s modulus of phase-change microcapsules (microPCMs) incorporated with different amounts of diatomite.

E/MPa	S0	S1	S2	S3	S4	S5
Maximum	1870.90	1970.20	2476.95	2874.94	3065.39	2583.59
Minimum	102.09	94.56	157.48	189.57	197.45	161.58
Average	575.49	589.43	768.23	903.26	942.85	773.05
